# TRASH: Tandem Repeat Annotation and Structural Hierarchy

**DOI:** 10.1093/bioinformatics/btad308

**Published:** 2023-05-10

**Authors:** Piotr Wlodzimierz, Michael Hong, Ian R Henderson

**Affiliations:** Department of Plant Sciences, University of Cambridge, Cambridge CB2 3EA, United Kingdom; Department of Genetics, University of Cambridge, Cambridge CB2 3EA, United Kingdom; Department of Plant Sciences, University of Cambridge, Cambridge CB2 3EA, United Kingdom

## Abstract

**Motivation:**

The advent of long-read DNA sequencing is allowing complete assembly of highly repetitive genomic regions for the first time, including the megabase-scale satellite repeat arrays found in many eukaryotic centromeres. The assembly of such repetitive regions creates a need for their *de novo* annotation, including patterns of higher order repetition. To annotate tandem repeats, methods are required that can be widely applied to diverse genome sequences, without prior knowledge of monomer sequences.

**Results:**

Tandem Repeat Annotation and Structural Hierarchy (TRASH) is a tool that identifies and maps tandem repeats in nucleotide sequence, without prior knowledge of repeat composition. TRASH analyses a fasta assembly file, identifies regions occupied by repeats and then precisely maps them and their higher order structures. To demonstrate the applicability and scalability of TRASH for centromere research, we apply our method to the recently published Col-CEN genome of *Arabidopsis thaliana* and the complete human CHM13 genome.

**Availability and implementation:**

TRASH is freely available at:https://github.com/vlothec/TRASH and supported on Linux.

## 1 Introduction

Separation of eukaryotic genomic DNA using centrifugation through density gradients revealed satellite bands with distinct %GC sequence content, relative to the rest of the genome (Thakur et al. 2021; Altemose 2022). Genome sequencing has revealed that satellite DNA frequently corresponds to large tandem repeat arrays (Thakur et al. 2021; Altemose 2022). This includes centromere satellite arrays, which act to mediate kinetochore formation and chromosome attachment to spindle microtubules during cell division (McKinley and Cheeseman 2016; Thakur et al. 2021; Altemose 2022). The centromeres of many eukaryotes consist of kilobases to megabases of tandem satellite repeats, where each monomer repeat is close to the length of DNA occupied by a single nucleosome (∼100 to 200 nucleotides), and which may also form higher order repeats (HORs) (Henikoff et al. 2001; Melters et al. 2013; Miga and Alexandrov 2021). Satellite repeats are proposed to duplicate, delete or change via replication slippage, unequal crossing-over and gene conversion (Rudd et al. 2006; Miga and Alexandrov 2021; Thakur et al. 2021; Altemose 2022). In addition to centromere satellite repeats, eukaryotic genomes contain diverse other classes of tandem repeats that perform multiple roles, including telomere repeats, ribosomal DNA, and mini- and micro-satellites (Thakur et al. 2021; Altemose 2022). Methods are thus required to robustly identify and annotate tandem repeats in nucleotide sequences.

Due to their extreme sequence repetition, it has been challenging to correctly assemble large tandem repeat arrays (Miga and Sullivan 2021). However, the advent of long-read DNA sequencing technologies, including Oxford Nanopore and PacBio HiFi, has allowed accurate and complete assembly of complex satellite arrays for the first time (Jain et al. 2018; Miga et al. 2020; Logsdon et al. 2021; Naish et al. 2021; Altemose et al. 2022; Nurk et al. 2022). The availability of these complete assemblies necessitates development of specific tools to identify and annotate tandem and other sequence repeats. A range of existing software exists for repeat annotation. For example, RepeatMasker uses Basic Local Alignment Search Tool (BLAST) and a library of transposable elements (Smit et al. 2014), Tandem Repeats Finder (TRF) uses an algorithm to *de novo* extract tandem repeat families (Benson 1999), and RepeatExplorer2 uses graph-based clustering to annotate repeats (Novák et al. 2010). Although these tools are effective for *de novo* identification of regions of tandem repeats, they do not precisely annotate individual repeat locations, or HORs.

More recently, specific tools have been developed to annotate the human centromeric α-satellite arrays and their higher order structures, including HORmon (Kunyavskaya et al. 2022), centroFlye (Bzikadze and Pevzner 2020), Alpha-CENTAURI (Sevim et al. 2016), HiCAT (Gao et al. 2022), and CentromereArchitect (Dvorkina et al. 2021). Although these tools are effective in human genomes, they rely on prior mapping of repeats and in some cases monomer definitions, understood as division of individual repeats into highly similar classes that define HOR subunits, which limits their wider applicability. Other software designed to annotate tandem repeats are also available, including PHOBOS that focuses on short repeats (1–50 bp) (Read et al. 2013), and TRAL that is designed to identify internal tandem repeat in proteins (Schaper et al. 2015). In summary, a method for annotation and analysis of megabase tandem arrays typical of eukaryotic centromeres, which does not rely on previously identified repeats, is required.

To meet this need, we designed Tandem Repeat Annotation and Structural Hierarchy (TRASH) to annotate tandem repeats in a genome sequence with no prior information of monomer or HOR structures. TRASH uses a fasta sequence file as input and outputs information on repeats, their clustering into families and HOR analysis. TRASH searches for continuous, highly similar, tandemly arranged DNA repeats of a similar unit size. This excludes transposable elements and interspersed repeats from analysis and allows for precise definition of tandemly arranged repeats. To provide validation of TRASH we report its performance and output on the *Arabidopsis thaliana* Col-CEN genome assembly (Naish et al. 2021). *Arabidopsis thaliana* is a monocentric model plant species with a relatively small genome (∼130 Mb), yet has megabase length tandem arrays of the *CEN178* satellite repeat associated with its centromeres (Naish et al. 2021), making it suitable genome for benchmarking of TRASH against other repeat identification software. We additionally benchmark TRASH against the complete human CHM13 genome, which contains large tandem repeat arrays (Altemose et al. 2022; Nurk et al. 2022). We show that TRASH is effective at defining tandem repeat monomers and their higher order structures in the larger (∼3054 Mb) CHM13 genome assembly.

## 2 Materials and methods

### 2.1 Tandem repeat region and consensus repeat identification

**Figure 1.**
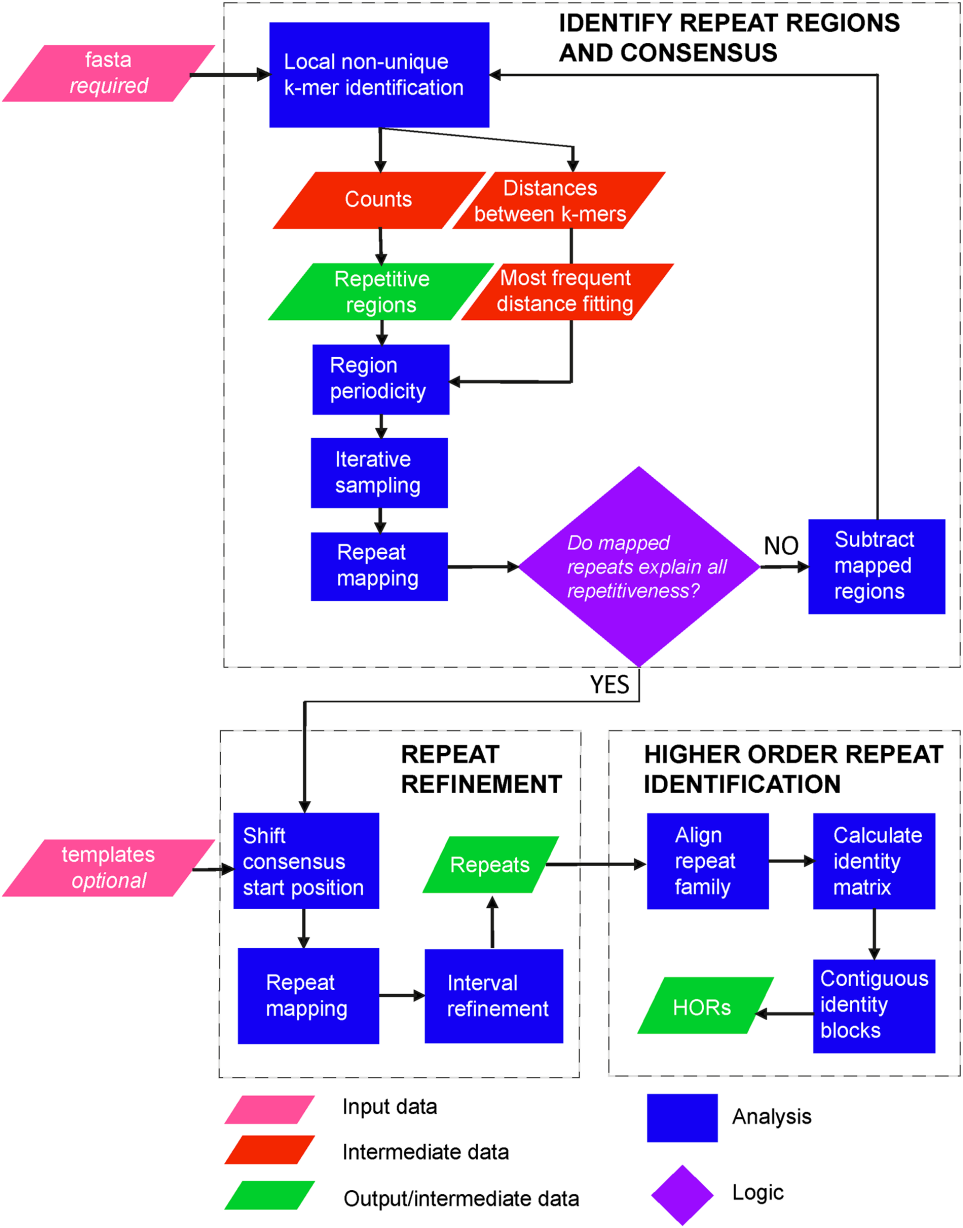
TRASH workflow for tandem repeat and HOR annotation. TRASH consists of three modules; (i) repeat region and consensus identification, (ii) repeat refinement, and (iii) HOR identification. For repeat identification, the only required input is a fasta sequence file. In addition, sequence templates can be provided to assign identified repeats to families and align their start positions (optional). When sequence templates are provided, repeats of the same family can be analysed for their higher order structures.

TRASH analyses an input fasta file, i.e. first divided into individual sequences, which are processed separately and in parallel. In the first step, each sequence is divided into windows of a set number of nucleotides (1500 base pairs by default) (Fig. 1). Each of these windows has a repeat content score assigned by calculating the proportion of non-unique k-mers relative to the window size (*k* = 10 nucleotides by default). These scores tend to approach 0% for windows without repeats, whereas values will be in the range of 80–100% for windows occupied by tandem repeats. Otsu’s method is used to find the optimal threshold that divides the bimodally distributed window scores into those containing repeats and those that do not (Supplementary Fig. S1A). All windows above this threshold are marked as repetitive, as they contain a high number of internally repeated k-mers, and those that are physically adjacent are concatenated. This results in a list of repetitive regions, to which subsequent analysis is restricted (Fig. 1).

When a region consists completely of tandem repeats of size *N*, then any random *N*-sized sub-sequence is likely to be representative of the entire region. Hence, the size of the individual repeat is estimated by measuring the distance between start coordinates of each pair of identical k-mers and finding the most common *N* value. This *N* value is used to sample the region a number of times (6 by default), to randomly extract *N* length subsequences. These are mapped back to the region using the matchPattern function from the R Biostrings package (Pages et al. 2023). The set of matches that covers the greatest part of the region is then extracted and aligned using MAFFT (settings: **–**kimura 1 **–**retree 1) to create a primary consensus sequence (Fig. 1) (Katoh and Standley 2013).

In some cases, distinct repeat families are found immediately adjacent to one another, or can be interspersed within a repetitive region. For these cases, TRASH checks the coverage of the primary consensus mapping, and if a continuous sequence of more than the allowed size remains, it splits the region. This newly created region enters the same process as described above, until no further repeats can be found, or no more sequence with unmapped repeats remains (Fig. 1).

When HORs exist within an array, they may prevent TRASH from correctly identifying the monomers, which may instead identify multiples of monomers as the base repeat. In such scenarios, the ‘**–**N.max.div’ flag can be used to split the repeats into monomers (Supplementary Fig. S1B and C). Using this method, TRASH divides the most frequent k-mer *N* by a range of integers (2–12 by default, with the upper limit controlled using the ‘**–**max.N.split’ flag) and checks whether peaks exist at the new k-mer distances. For each integer *d* (2–12 by default), TRASH will sum k-mers found surrounding the *N*/*d* distance and take the highest possible *d*, i.e. above a percentage threshold set by the user. This threshold, controlled with the ‘**–**N.max.div’ flag, is set at 100 by default, meaning the method is normally not functional. When considering composite numbers (4, 6, 8, 9, etc.), TRASH will also consider the number of k-mers around distance values that correspond to division of *N* by all the positive divisors (other than 1 and itself).

### 2.2 Tandem repeat refinement

Since repeats within tandem arrays do not have intrinsic start or end positions, it can be challenging to compare those from the same family but mapped in a different shift. For example, when considering the sequence ‘ACTACTACTACTA…’, either ‘ACT’, or ‘CTA’ are valid representatives of the base repeat, which when aligned produce a ‘-CT-’ consensus sequence with two gaps. However, if all repeats are shifted to the same start, ‘CTA’, then alignment produces a gapless ‘CTA’ consensus. Furthermore, repeats can be identified on either the forward or reverse strands. We therefore process the primary consensus to change its start position and strand. The sequence of the primary consensus is divided into *n* 6-mers starting at each position, with the last k-mer taking nucleotides from the beginning of the sequence. Then, a hash function assigns a unique 6-mer score *K*: where *V_i_* is a nucleotide-specific value (A = 0, C = 1, T = 2, G = 3), and *i* is the position of that nucleotide within the 6-mer. Any sequences containing non-standard nucleotides are removed from analysis at an earlier stage.


$$K=\sum_{i=1}^{6}V_{i}4^{i}$$

The shift score *S* is then calculated using the list of *K* scores:


$$S=\sum_{j=1}^{n}jK_{j}$$

**Figure 2.**
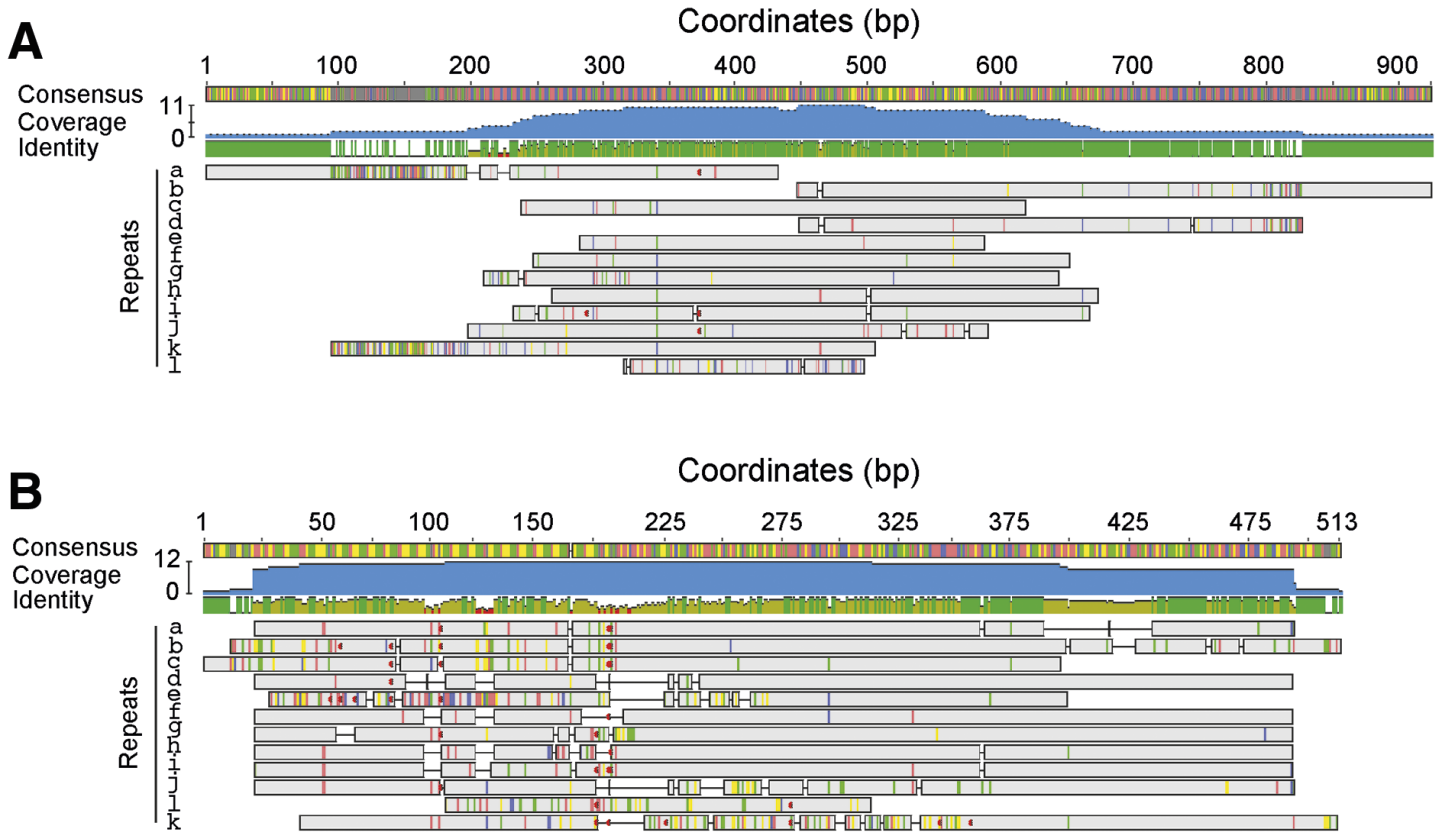
Shifting the frame of related repeats using TRASH. (A) A multiple pairwise sequence alignment of 12 *5S* rDNA repeats from the Col-CEN assembly is shown, prior to shifting the repeat consensus. Variable start positions cause uneven alignment coverage, making sequence comparisons less accurate and causing ‘end gaps’ when coverage against the consensus is plotted. (B) A multiple sequence alignment of the same *5S* rDNA repeats is shown, following shifting the repeat start positions via TRASH. This causes repeats to have increased and more even alignment coverage, which allows for more accurate comparison of the repeats.

The same is done for every possible shift of the primary consensus and the reverse complement, and the shift with the lowest score is replaced as the primary consensus. This secures similar shifts for related repeats. For example, we show the results of alignment of a group of *5S* rDNA repeats from *A.thaliana* with and without shifting (Fig. 2).

In addition to functioning in *de novo* mode, when prior information on the repeat families populating the analysed sequence exists, this can be provided to TRASH in the form of a table with the name and consensus sequence of the repeat, termed a ‘template’ (Fig. 1 and Supplementary Table S1). This optional step allows classification of identified repeats into families. Repeat family classification is used for HOR mapping, which requires that only the same family of repeats is analysed (Fig. 1). When provided, each primary consensus is compared against each template using a k-mer based, alignment free, identity calculation. Briefly, query and template sequences are divided into 8-mers in a sliding window, with the last 8-mers using nucleotides from the beginning of the sequence. The two sets of 8-mers are compared and the Jaccard similarity index is returned as a similarity score. The name of the template with the highest score is assigned to the ‘family name’ parameter of the region. Alignment scores are also tested for significance, at a 95% confidence level, by comparing the similarity score to the distribution of scores from random permutations of the query sequence. The primary consensus is then once again adapted to correspond to the specific shift of the template sequence. This is done by creating all possible shifts of the primary consensus, aligning it to the template sequence, and extracting the shift with the highest score.

Having adjusted the primary consensus sequence, a second mapping step is performed using matchPattern, resulting in a new list of repeats (Fig. 1). A secondary consensus sequence is derived as before, and a polishing step is applied where any short overlap between neighbouring repeats, defined as less than half the length of the secondary consensus, is divided equally and both overlapping repeats are shortened. In the case of long repeat overlaps, defined as more than, or equal to, half the length of the secondary consensus, the shorter of the two repeats is removed. For short gaps between repeats (up to 4 nucleotides by default) the start and end coordinates of surrounding repeats are shifted to fill those gaps evenly. When repeat templates are provided, any classified repeats in the output are aligned and a consensus sequence is generated. From this, the Levenshtein edit distance between the consensus and each individual repeat monomer is calculated and reported in the output file.

### 2.3. Higher order repeat (HOR) identification

As the DNA alphabet has now been translated into repeats, the repeat alphabet can be translated into HORs. Available HOR identification software rely on the existence of discrete sub-classes of repeats called monomers (Dvorkina et al. 2020), monomer subtypes (Altemose et al. 2022), or basic repeat units (Koga et al. 2014). For example, each repeat can be assigned a monomer identity based on similarity levels to predefined monomer classes, which allows translation of repeats into a string of monomers and HOR identification via string decomposition (Dvorkina et al. 2020). However, if repeat similarity levels do not allow for faithful division into subclasses, HORs may not be robustly identified. The TRASH HOR identification module does not require monomer class definition, which eliminates this potentially problematic step.

For identification, a HOR is understood as a pair of repeat blocks, each consisting of the same number of monomers, with monomer pairs occupying the same relative within-block position having high similarity. Repeat similarity is measured as the number of disagreements between the two repeats in the multiple-sequence alignment of all analysed repeats, termed the variant score (*VS*). Two parameters are therefore considered in the identification of HORs: the minimal length of monomers per repeat block, termed *LM*, and the maximum *VS*, termed *VS*^max^. First, the repeats from an array(s) of interest are used for a multiple sequence alignment, performed using MAFFT (settings: –kimura 1 –retree 2) (Katoh and Standley 2013). For each pair of repeats within the alignment, their *VS* is calculated, which is considered in a triangular matrix (Supplementary Fig. S2). The off-diagonals of the matrix are traversed and if *VS* ⩽ *VS*^max^ is encountered, a new HOR instance is created (Supplementary Fig. S2). The HOR can be extended by testing the consecutive *VS* scores of adjacent monomers, until one higher than *VS*^max^ is found. If at least *LM* monomer pairs meet the criterion, the HOR is reported. This identifies HORs in which two blocks are on the same DNA strand. For HORs in which blocks are on the opposite DNA strands, the parallels to the diagonal of the matrix are traversed in the same manner. Individual monomers can be a part of multiple HORs and blocks of an individual HOR can overlap. By default, TRASH uses *LM *=* *3 and *VS*^max ^= 5, which can be adjusted when more or less stringent identification is required. To quantify the involvement of each monomer in higher order structures, we apply a repetitiveness metric that sums the lengths (in monomers) of HORs that a repeat is involved in (Supplementary Figs S2 and S4).

**Figure 3.**
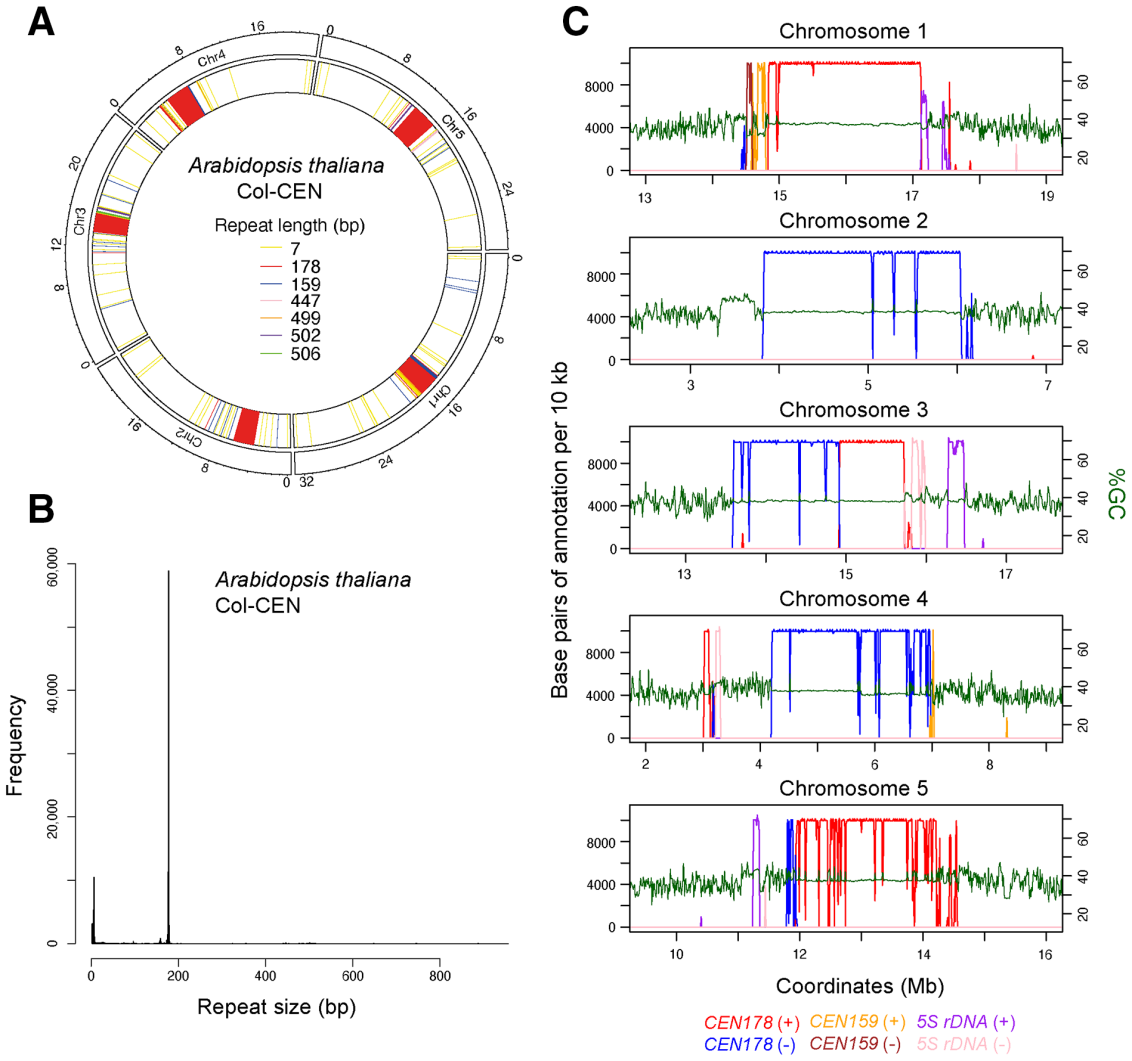
Tandem repeat identification in the *A.thaliana* Col-CEN genome by TRASH. (A) A circos plot of tandem repeats identified by TRASH in *de novo* mode in the *A.thaliana* Col-CEN genome assembly (Naish et al. 2021), which is a default output of the TRASH repeat identification module. Shading is coloured according to repeat length (bp). (B) Histogram of tandem repeat lengths (bp) identified in Col-CEN by TRASH in *de novo* mode. Visible peaks correspond to telomeric (7 bp), *CEN159* (159 bp), and *CEN178* (178 bp) repeat families. (C) Plots of the five Col-CEN centromeric regions showing %GC base content in 10 kb windows (green), together with TRASH annotation of tandem repeats. The number of bases of repeat annotation are plotted per 10 kb window, with *CEN178* centromere satellites on forward (red) and reverse (blue) strands, *CEN159* on forward (orange) and reverse (brown) strands, and *5S* rDNA on forward (purple) and reverse (pink) strands shown.

**Figure 4.**
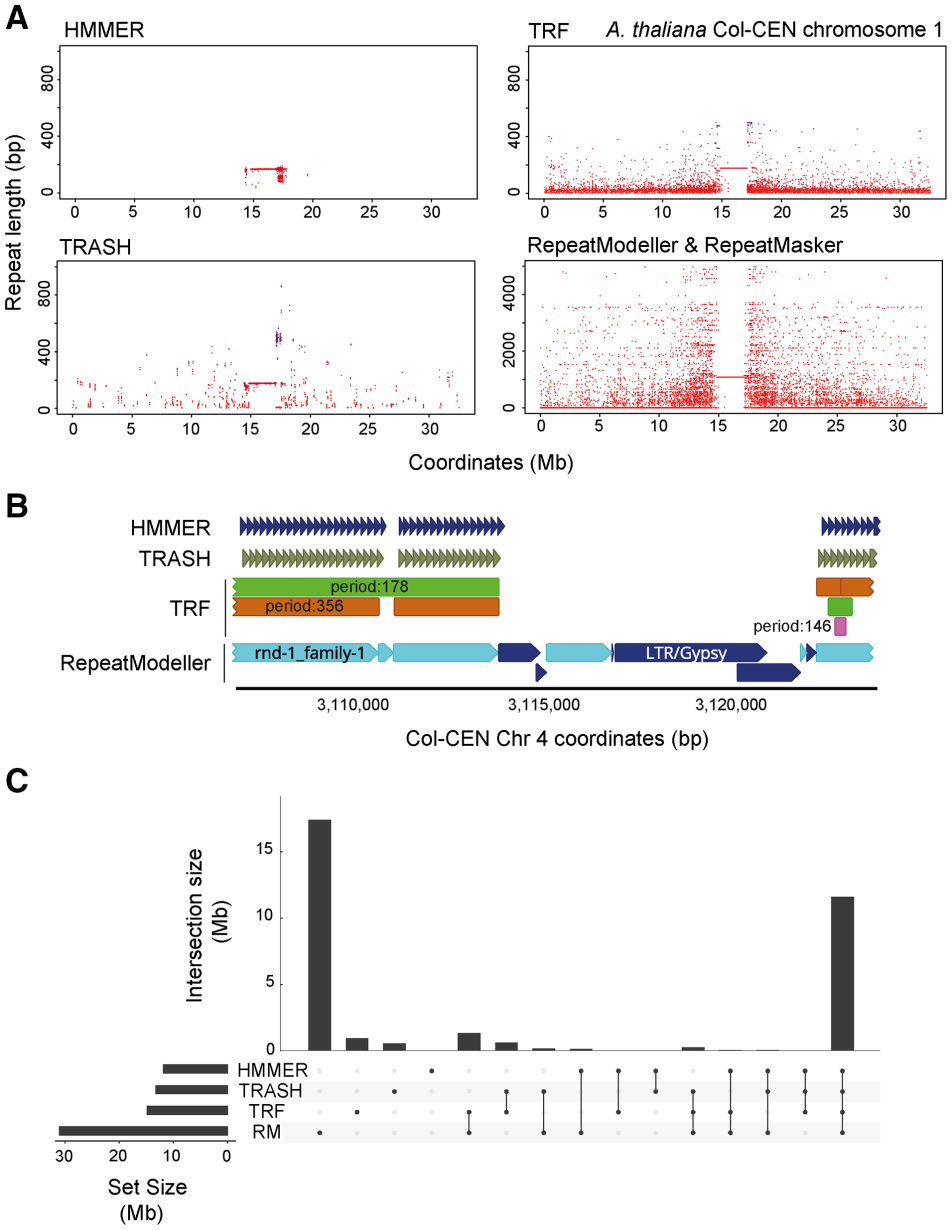
Benchmarking TRASH against alternative software for *de novo* tandem repeat identification. (A) Tandem repeats (red) plotted along chromosome 1 of the Col-CEN assembly identified by TRASH run in *de novo* mode, Tandem Repeat Finder (TRF), and RepeatModeller with RepeatMasker, or by aligning a *CEN178* satellite consensus using HMMER. The *y* axis represents repeat unit length (bp). (B) An example region of *A.thaliana* chromosome 4 (3 106 910–3 123 586 bp) showing repeat annotations generated by TRASH in *de novo* mode, RepeatModeller, TRF, and HMMER. The TRF annotation includes information on the periodicity of the annotated regions. ‘rnd-1_family-1’ corresponds to a repeat family of size 1054 bp from the RepeatModeller library output. (C) An upset plot showing overlaps of base-pair coverage of *A.thaliana* repeats *de novo* identified with TRASH, TRF and RepeatModeler, and *CEN178* consensus-based alignment with HMMER. The adjacent Set Size plot shows the total base pairs of repeats (Mb) identified by each method. The Intersection Size plot shows the total base pairs (Mb) of repeats that are uniquely annotated by each software, or combinations of software. The software being considered are indicated by the black dots beneath.

**Figure 5.**
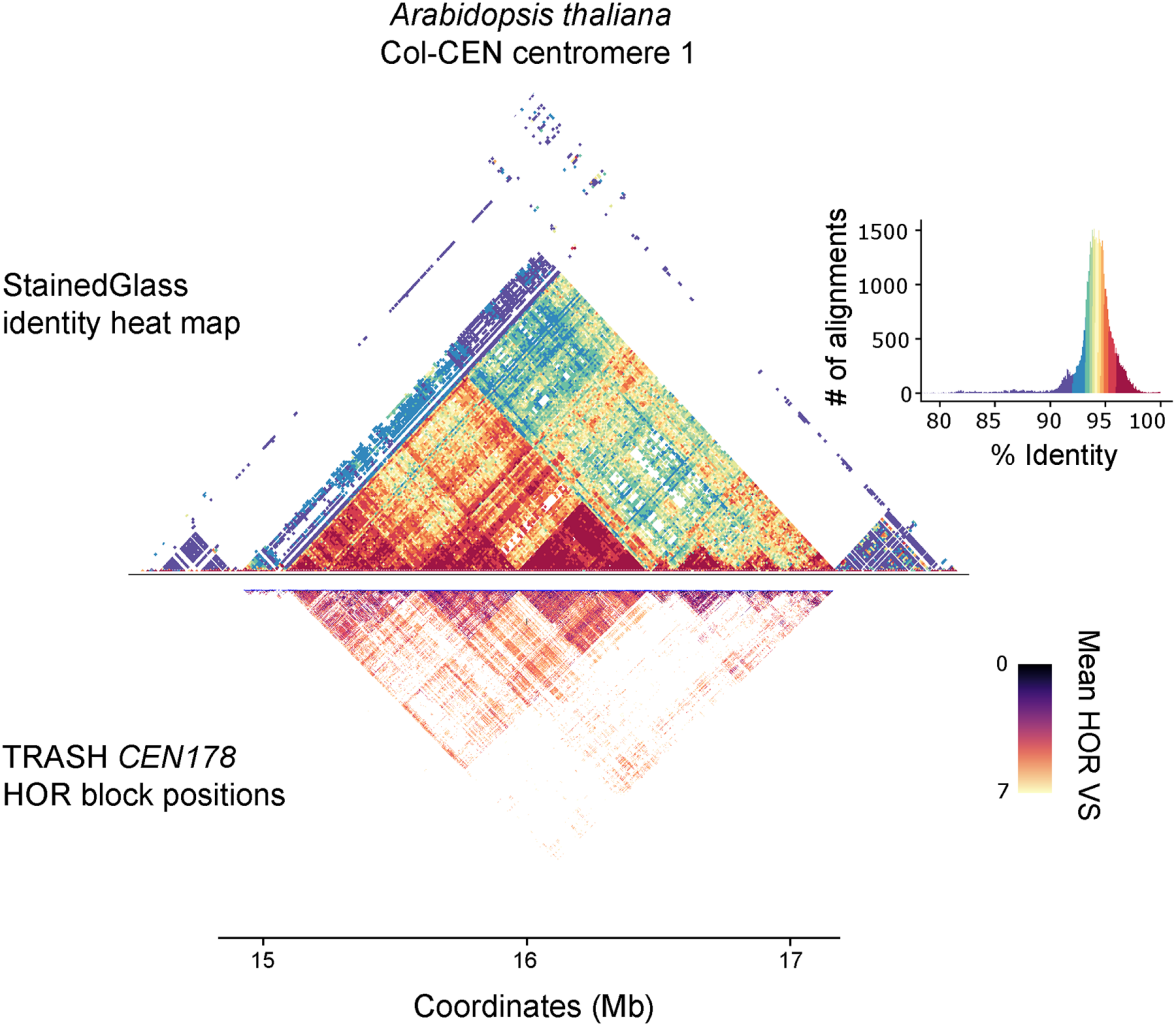
HOR analysis in Arabidopsis centromeres using TRASH. A StainedGlass sequence identity heat map of *A.thaliana* centromere 1 (14 442 038–17 870 129 bp) from the Col-CEN assembly is shown (Vollger et al. 2022). A StainedGlass histogram showing assignment of colours to sequence identity values is shown alongside. Beneath is a heat map showing the positions of *CEN178* HORs identified by TRASH over the same region, with shading according to the mean variant score (*VS*) between pairs of HORs. The HOR search was performed using TRASH default settings and the *CEN178* consensus repeat as the template.

To facilitate analysis of results, TRASH outputs are visualized using a circos plot that represents the position of most abundant repeat families identified, plotted against the DNA sequence analysed (Fig. 3A). In addition, a linear plot of each sequence that represents all identified repeats and their sizes can be produced (Fig. 4A and Supplementary Fig. S3). When HOR identification is performed, a dot plot representing start locations of HOR blocks can be generated (Fig. 5 and Supplementary Fig. S4), which can be compared to the repetitiveness metric to visualize HOR patterns.

## 3 Results

### 3.1 *Arabidopsis thaliana* Col-CEN assembly

To validate TRASH, we analysed the *A.thaliana* Col-CEN genome (Supplementary Table S2) (Naish et al. 2021). Using an 8 CPU 40 GB RAM machine, the runtime using *de novo* default settings was under 3 h. In addition, we ran TRASH using sequence templates that represent previously known *A.thaliana* tandem repeats (Supplementary Table S1). For *CEN178* HOR identification we used the settings ‘-seqt templates.csv -horclass cen178’. Tandem Repeat Finder (version 4.0.9) benchmarking analysis was performed using the recommended settings; ‘2 7 7 80 10 50 500 -f -d -l 5’. RepeatModeller (version 4.1.2) was used to generate a repeat library using default settings, and RepeatMasker (version 4.1.2) was used with the resulting library output to generate a list of repeats. As an alignment-based approach, HMMER (version 3.3.2) was used to search for *CEN178* repeats using the nhmmer function with default settings, using a previously published *CEN178* consensus repeat as the query (Maheshwari et al. 2017). Several HOR identification pipelines exist, e.g. HORmon (Kunyavskaya et al. 2022), centroFlye (Bzikadze and Pevzner 2020), Alpha-CENTAURI (Sevim et al. 2016), HiCAT (Gao et al. 2022), and CentromereArchitect (Dvorkina et al. 2021). These methods generally use monomer subclass identification to derive one-dimensional HOR definitions. In contrast, TRASH HOR output is a list of multi-monomer duplications and for this reason, direct comparison between outputs on the Col-CEN was not performed.

TRASH identified a total of 96 793 tandem repeats in the Col-CEN genome, corresponding to 13 257 524 bp, or 10.03% of the 132 081 078 bp genome. Three sequence templates were provided for the run: *CEN178* (76 711 repeats identified), *CEN159* (1754 repeats identified), and *5S* rDNA (1261 repeats identified) (Supplementary Table S1). The histogram of repeat sizes identified by TRASH shows a single dominant peak that corresponds to the centromeric *CEN178* satellite repeats (∼178 bp) (Fig. 3B) (Naish et al. 2021). The lower abundance *CEN159* family was also observed, as well as 7 bp telomeric repeats (Fig. 3B). A *de novo* run that was performed without providing the sequence templates identified an almost perfectly overlapping set of repeats (99.99% Jaccard similarity index), demonstrating the effectiveness of TRASH for *de novo* tandem repeat definition. To facilitate alignment and other downstream analysis, the repeats of each family were identified with the same shift, further highlighting the utility of TRASH for accurate annotation.

To provide an independent means of benchmarking TRASH against other *de novo* repeat annotation software, we used an alignment based method. For example, if a repeat consensus is known, alignment tools such as BLAST or HMMER can be used to identify repeat copies (Altschul et al. 1990; Wheeler and Eddy 2013). Therefore, we used HMMER with a previously published *CEN178* consensus sequence to map repeats (Maheshwari et al. 2017). TRASH was able to identify repeats that overlapped with 98.29% of the total coverage identified using HMMER (Fig. 4C).

To perform benchmarking, we compared the TRASH annotation to results from alternative software; Tandem Repeats Finder (TRF) (Benson 1999), and RepeatModeller (Flynn et al. 2020), followed by RepeatMasker (Tempel 2012). Other repeat methods, including TRAL and PHOBOS, were not applicable for direct comparison with TRASH, due to limitations on repeat size detection and input sequence length. Identified repeats and their monomer length were plotted from each software (Fig. 4A). All three *de novo* methods identified long arrays of *CEN178* repeats corresponding to the centromere locations, in addition to many repeats of varying length throughout the chromosome arms (Fig. 4A). In addition, RepeatModeller was able to identify repetitive elements that are not tandemly arranged, including transposable elements (Flynn et al. 2020). Although each method accurately identifies tandem repeat regions, only TRASH *de novo* identified and annotated individual repeats in the arrays, with an added benefit of being in the same shift (Fig. 4A and B). In contrast, TRF identified 78% of *CEN178* repeats in a monomer state, and the remaining repeats as dimers. RepeatModeller did not have a *CEN178* template annotated in its library and identified repeats as sextuples (1054 bp) of the 178 bp repeat. In addition, in both RepeatModeller and TRF outputs, *CEN178* repeats are not individually mapped, but rather annotated as regions of repeats, together with information on the underlying monomers, which makes it challenging to extract individual repeats for downstream analysis (Fig. 4B). Nonetheless, when HMMER derived *CEN178* positions were considered, each *de novo* method was able to identify >98% of their total coverage (Fig. 4C).

We used an upset plot to visualize overlap between repeats identified by each method (Fig. 4C). Each position in the Col-CEN assembly was checked as to whether it was identified by each software, and the number of positions identified by the same method, or group of methods, were plotted (Fig. 4C). This showed that 11.5 Mb of repeats were identified by all four approaches, representing the main *CEN178* arrays. A notable set of positions identified solely by RepeatModeller represent transposable elements and other non-tandemly arranged repeats.

Overall, TRASH can identify tandem repeat regions and accurately map individual monomers, providing this information in a tabulated form that facilitates downstream analysis. The ‘repeats’ file generated contains the coordinates of all identified repeats with their sequence, and if sequence templates were provided, repeat family and edit distance against the sequence-wide consensus are also given (Fig. 1 and Supplementary Table S1). If the HOR module was used, their repetitiveness score is provided (Supplementary Table S3). HORs are reported as a table of block duplications, with the sum of *VS* between each pair of repeats included (Supplementary Fig. S2 and Supplementary Table S3). A repetitiveness metric is also reported per monomer repeat, which is the sum of the lengths (in monomers) of HORs that a repeat is involved in. In addition, circos or linear plots are generated to provide an overview of repeat distributions along the analysed DNA sequences (Figs 3 and 4 and Supplementary Figs S3 and S4).

The main disadvantage to TRASH is that it is restricted to detecting continuous arrays of tandem repeats. Interspersed repeats, including transposable elements, will not be annotated, unless they are themselves arranged in tandem arrays. Similarly, when repeats are interspersed by short, but unrelated sequences, TRASH might concatenate the repeated element together with inserts. For example, sequence {*A*_1_*B A*_2_*C A*_3_*D*} might be identified as a series of *A*_n_{*B*/*C*/*D*} repeats.

We used the TRASH repeat output and selected the *CEN178* satellite repeat family for HOR analysis, using default settings. In total, 9 795 284 HOR instances were identified in the *A.thaliana* Col-CEN assembly (Fig. 5, Supplementary Fig. S4 and Supplementary Table S3). To visualize the TRASH HOR output, the identified HORs can be plotted in a two-dimensional dot plot matrix (Fig. 5 and Supplementary Fig. S4). We compared TRASH HOR annotation to a StainedGlass sequence identity heat map of the same sequence (Vollger et al. 2022). StainedGlass is a visualization method that divides the genome into windows, scores them for pairwise similarity and plots a heat map of the resulting values (Fig. 5) (Vollger et al. 2022). Consistently, regions with higher pairwise similarity identified by StainedGlass correspond to regions that TRASH annotated with abundant *CEN178* HORs (Fig. 5).

### 3.2 *Homo sapiens CHM13 genome assembly*

To further benchmark TRASH performance, we *de novo* analysed the ∼3054 Mb human CHM13 genome (Altemose et al. 2022; Hoyt et al. 2022; Nurk et al. 2022). Human chromosomes feature large tandem repeat arrays that in total comprise ∼6.2% of the genome (Altemose et al. 2022; Hoyt et al. 2022; Nurk et al. 2022). The centromeres are the site of CENP-A nucleosome loading and are located within megabase α-satellite arrays, consisting of ∼170 bp monomers, that show extensive higher order repetition (Altemose 2022; Altemose et al. 2022; Hoyt et al. 2022; Nurk et al. 2022). The α-satellite arrays are flanked by more diverged regions of β- and γ-satellites, which show a greater degree of sequence interruption (Altemose 2022; Altemose et al. 2022; Hoyt et al. 2022; Nurk et al. 2022). In addition, human centromeric and pericentromeric regions contain large tandem arrays of the HSat1A, HSat2, and HSat3 satellites, which typically have shorter monomer sizes (5 to 42 bp) (Altemose 2022; Altemose et al. 2022; Hoyt et al. 2022; Nurk et al. 2022).

We analysed the CHM13 assembly using TRASH in *de novo* mode and compared the results to published tandem repeat annotations (Altemose 2022; Altemose et al. 2022). The sequences of the 24 CHM13 chromosomes were divided into 88 extractions to allow TRASH to run in parallel on multiple CPU cores. The *de novo* TRASH run analysing CHM13 used 88 cores of Intel Xeon Platinum 8276 CPU @ 2.20 GHz with 82 GB of allocated RAM and took 11 h and 24 min. While analysis of most sequences completed within the first 2–3 h of runtime, the presence of >10 Mb HSat arrays resulted in extension of the runtime to over 10 h.

**Figure 6.**
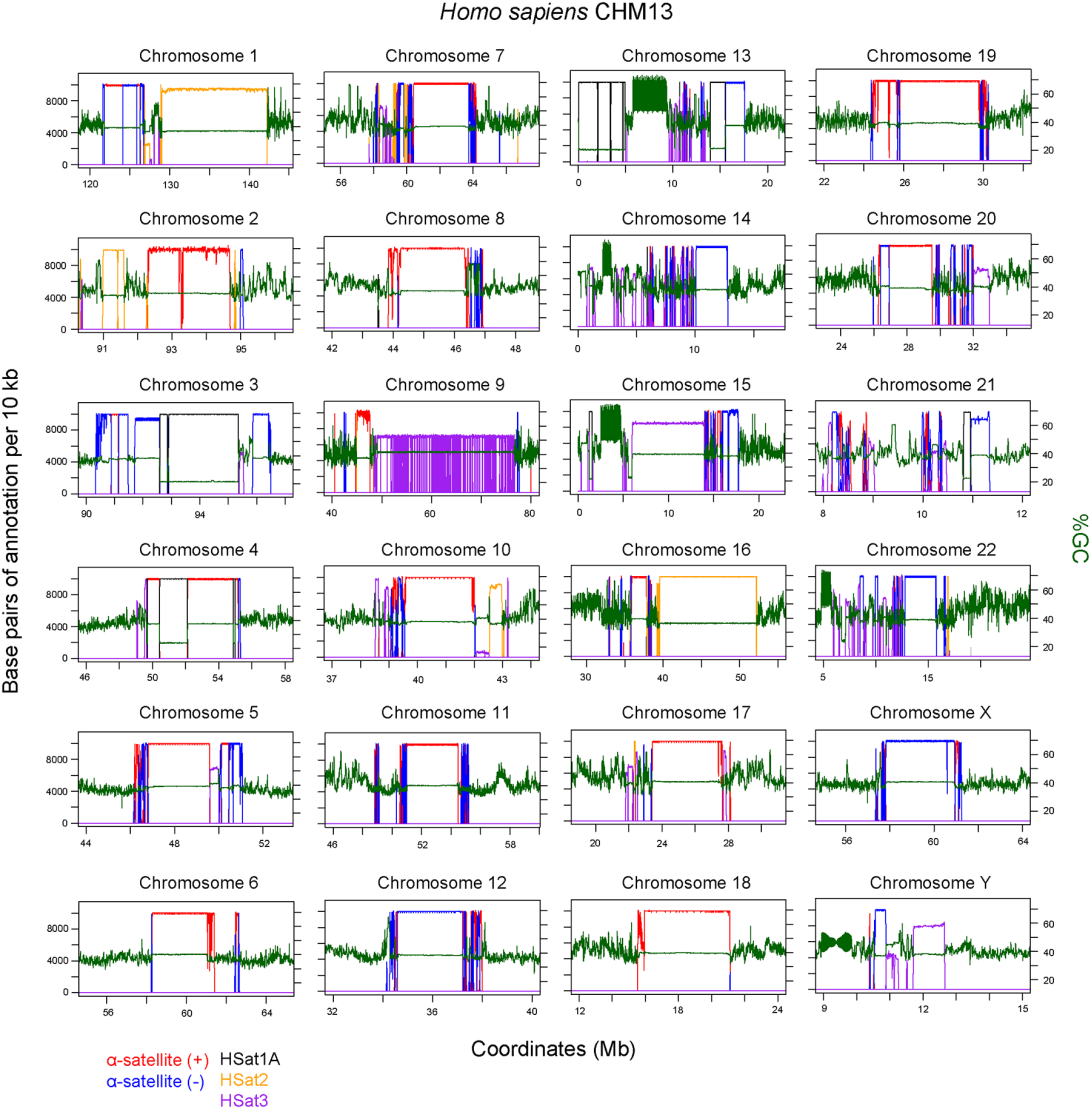
TRASH annotation of the human CHM13 genome in the centromeric regions. Plots of the 24 centromeric regions from the complete human CHM13 genome (Altemose et al. 2022; Nurk et al. 2022), showing %GC base content in 10 kb windows (green), together with TRASH *de novo* annotation of tandem repeats. Default settings were used by TRASH, except for chromosomes 2, 9, 15 and 21, which were run using the ‘–N.max.div 5’ setting to ensure α-satellite monomers were identified. The number of bases of repeat annotation per 10 kb window are plotted, with α-satellite centromere satellites on forward (red) and reverse (blue) strands, and HSat1A (black), HSat2 (orange), and HSat3 (purple) positions shown.

TRASH performed well identifying the major centromeric α-satellites and found repeats overlapping 96.2% of the regions reported (Fig. 6 and Supplementary Table S4) (Altemose 2022; Altemose et al. 2022). The β- and γ-satellites occur at lower copy numbers, are more divergent, and the arrays are more interrupted by other sequences (Altemose 2022; Altemose et al. 2022). As a consequence, TRASH found fewer of annotated β- and γ-satellites, with an overlap of 56.5% and 77.2%, respectively (Fig. 6 and Supplementary Table S4). The HSat1A, HSat2 and HSat3 satellite families have short monomer lengths (5 to 42 bp) and occur in megabase tandem repeat arrays (Altemose 2022; Altemose et al. 2022). TRASH successfully identified the majority of their annotated copies, with overlaps of 96.5%, 90.8%, and 73.4%, respectively (Fig. 6 and Supplementary Table S4). We confirmed that histograms of the repeats identified by TRASH that overlapped α-, β-, γ-satellite, HSat1A, HSat2, and HSat3 showed the expected size distributions (Supplementary Fig. S5). We calculated %GC content in 10 kb windows across the CHM13 genome, which allows tandem repeat array positions to be visualized as stretches of uniform GC content (Fig. 6). This confirmed that TRASH identified the major centromere-proximal tandem repeat arrays in CHM13 (Fig. 6).

We observed that repeats from the α-satellite arrays on CHM13 chromosomes 2, 9, 15, and 21 were annotated by TRASH as monomer multiples (Supplementary Table S5). For example, using TRASH *de novo* with default settings identified the most frequent k-mer distance on chromosome 2 (92 333 544:94 673 023) as 680 bp, representing a multiple of four 170 bp α-satellite monomers (Supplementary Fig. S1B and C). Using the setting ‘–N.max.div 5’, TRASH was able to split and identify the correct α-satellite period of 170 bp, which resulted in an annotation of 13 694 repeats (Supplementary Fig. S1B and C). Hence, TRASH annotation of chromosomes 2, 9, 15, and 21 was repeated using the ‘–N.max.div 5’ setting.

**Figure 7.**
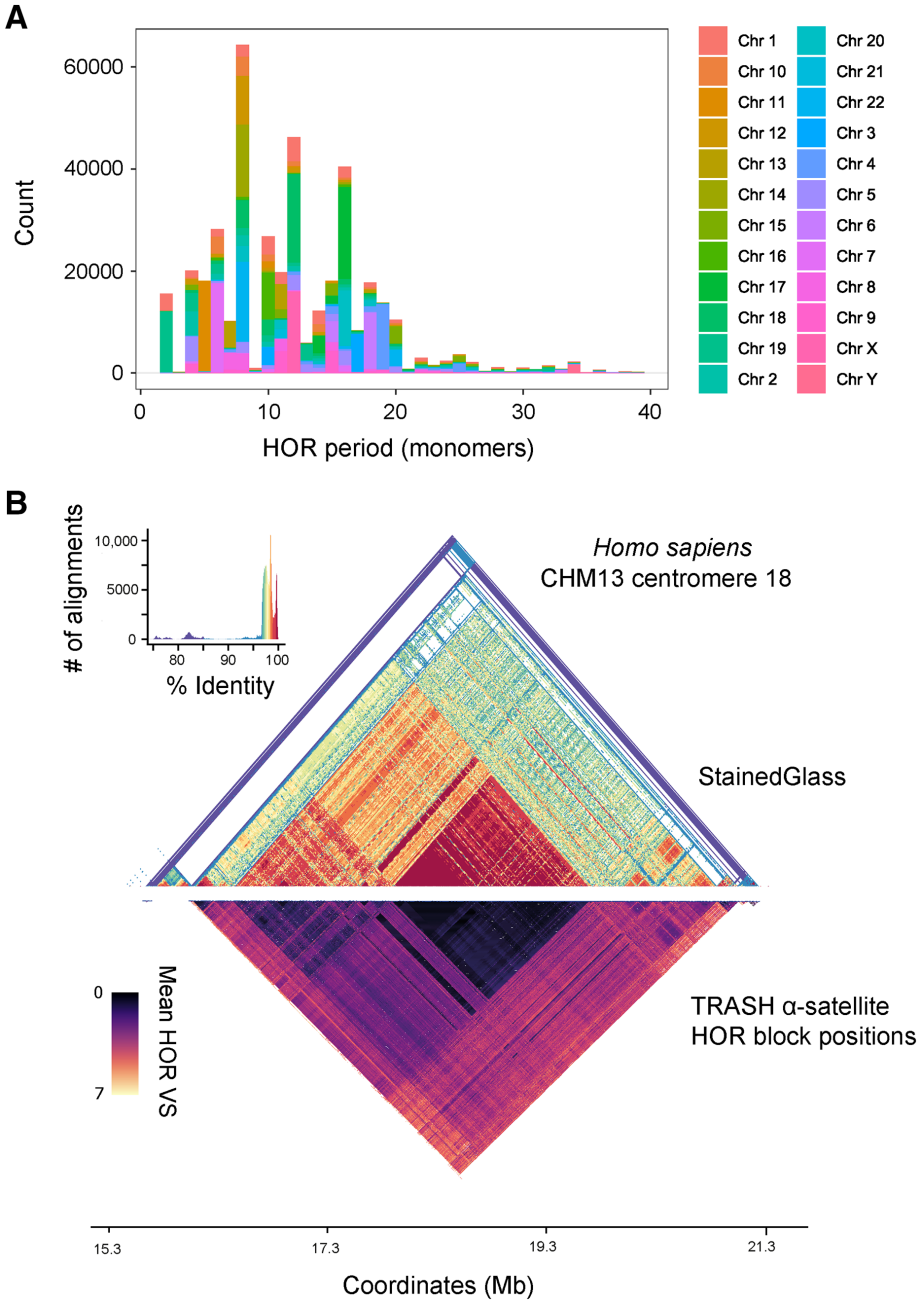
Analysis of α-satellite HORs in the CHM13 genome using TRASH. (A) Following the definition of CHM13 tandem repeats using TRASH in *de novo* mode, we selected repeats in the size range of α-satellite (160–180 bp) and performed HOR analysis using the default settings. For each chromosome, we calculated the predominant HOR periods within the α-satellites and plotted their counts, with colouring indicating chromosomes. (B) A StainedGlass sequence identity heat map of human centromere 18 from the CHM13 assembly is shown (Vollger et al. 2022). A StainedGlass histogram showing assignment of colours to sequence identity values is shown alongside. Beneath is a heat map showing the positions of α-satellite HORs identified by TRASH over the same region, with shading according to the mean variant score (VS) between pairs of HORs.

The α-satellites correspond to the site of kinetochore loading on human chromosomes and are documented to contain abundant HORs (Altemose et al. 2022; Nurk et al. 2022). We therefore sought to compare how TRASH performed when identifying α-satellite HORs in CHM13. Since the CHM13 TRASH run was performed in *de novo* mode, repeat classes were not automatically assigned. Therefore, we used all monomer repeats in the canonical size range for α-satellites (160–180 bp) to search for HORs. In total, 33 785 403 α-satellite HOR instances were identified in the CHM13 assembly (Fig. 7A and Supplementary Table S5). For each α-satellite, we identified the HOR with the shortest distance between its blocks and saved that distance (in monomers) as a proxy for the HOR period. For 17 of 24 chromosomes analysed, TRASH identified the same HOR period as reported (Fig. 7A and Supplementary Table S5) (Altemose 2022; Altemose et al. 2022). To visually display α-satellite HOR patterns annotated by TRASH, we compared with a sequence identity heat map of CHM13 centromere 18 analysed by StainedGlass (Fig. 7B) (Vollger et al. 2022). The TRASH heat map shows HOR block positions that are coloured according to the mean *VS* level (mean number of monomer variants between HOR monomers) (Fig. 7B). As for the Arabidopsis Col-CEN genome (Fig. 5), TRASH is effective at annotating HOR patterns that closely reflect patterns of sequence identity within CHM13 centromere 18.

We considered the remaining chromosomes, where TRASH identified a different HOR period to that reported (Fig. 7A and Supplementary Table S5) (Altemose 2022; Altemose et al. 2022). For chromosome 1, TRASH defined a HOR period of 12, which is twice that reported (6). On chromosome 5, the reported HOR period is 6, while TRASH defines a combination of interspersed 4, 8, 12, and 16 periods. On chromosome 8, the reported HOR period is 11, while TRASH defines periods of 7, 8, 11, and 15. On chromosome 9, the reported HOR period is 9, while TRASH defines periods of 11 and 15. On chromosome 13, the HOR period is reported as 11, while TRASH annotates an interspersed mixture of 7 and 11 periods. On chromosome 15, the reported HOR period is 11, while TRASH defines the most common period of 20 over the inactive ‘hor_15_3’ array, whereas the active ‘hor_15_4’ array is defined by TRASH as consisting of interspersed periods of 15 and 11. On chromosome 19, the reported HOR period is 6, while TRASH defines a period of 2, with secondary periods suggesting duplication (4) and triplication (6), of a base dimer. We conclude that in the majority of cases TRASH identified α-satellite HOR periods consistent with published annotation (Altemose 2022; Altemose et al. 2022).

## 4 Summary

TRASH can robustly and rapidly identify tandem repeat arrays in *A.thaliana* and human chromosomes, including the centromere satellite arrays and their higher order structures. Importantly, TRASH does not require any prior information about repeat families to operate. This means it will have wide utility for tandem repeat annotation as further uncharacterized telomere-to-telomere genome assemblies are generated across eukaryotes.

## Supplementary Material

btad308_Supplementary_Data

## Data Availability

T RASH output files from the Arabidopsis Col-CEN and human CHM13 genomes are available in the figshare repository at 10.6084/m9.figshare.22250326, 10.6084/m9.figshare.22250209, 10.6084/m9.figshare.22250185, and 10.6084/m9.figshare.22250191.
