# Supplementary material for: TRASH: Tandem Repeat Annotation and Structural Hierarchy
Source: Bioinformatics. 2023 May 10;39(5):btad308. doi: 10.1093/bioinformatics/btad308 (PMC10199239; doi:10.1093/bioinformatics/btad308)
Supplement: btad308_Supplementary_Data [file btad308_supplementary_data.docx]

**Supplementary Figures:**

**
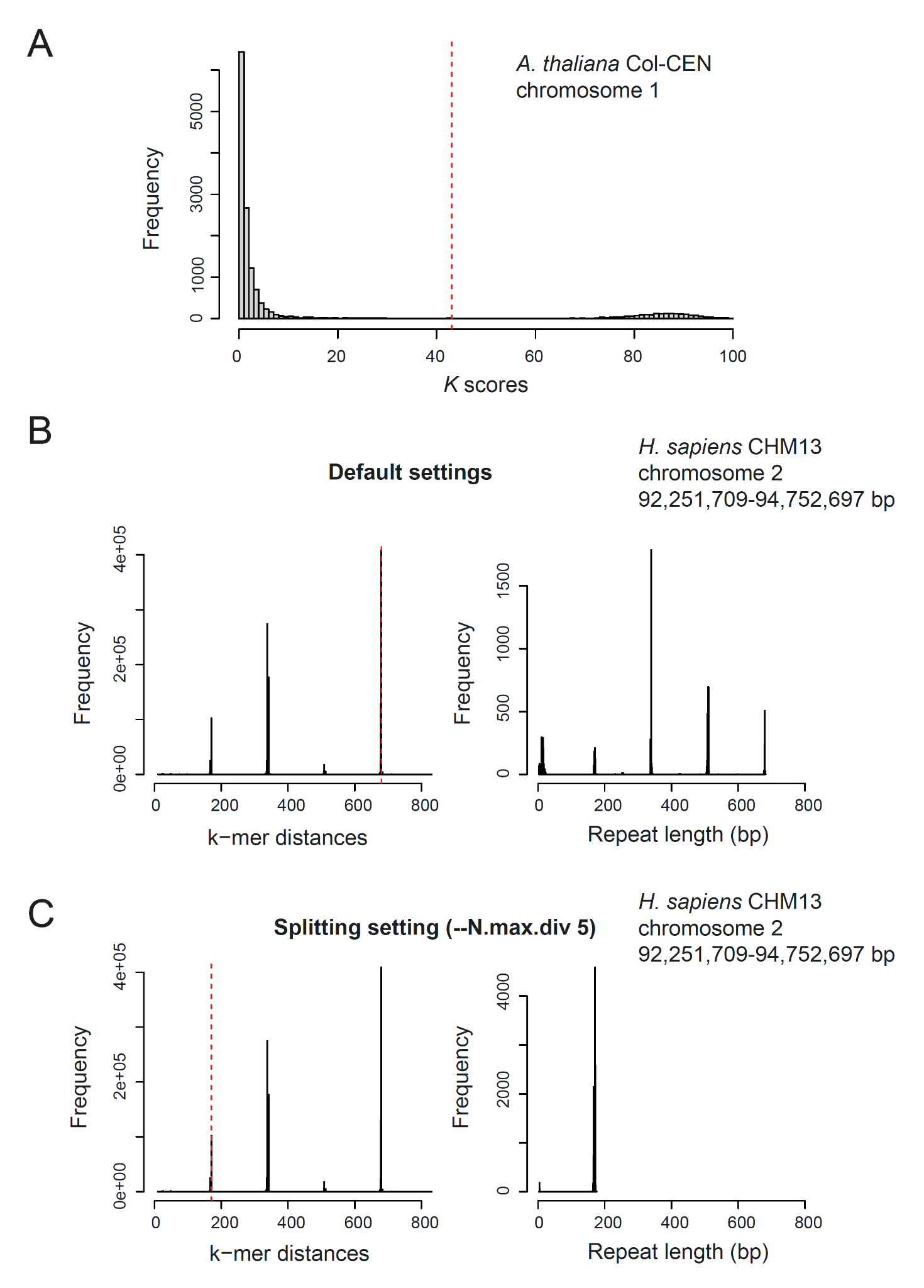
**

**Supplementary Figure 1. Identifying repetitive regions and monomer repeats using TRASH. A.** A histogram of the window repeat content score (*K*) in 10 kb windows within *A. thaliana* Col-CEN chromosome 1. The *K* score measures the frequency of repetitive k-mers per window. Windows were divided into those containing repeats, and those that do not, using Otsu’s threshold, which is indicated by the dotted red line. **B.** TRASH analysis of human CHM13 chromosome 2 in *de novo* mode using default settings was performed, which identified the most frequent repeat k-mer distance as 680 bp, which is multiple of four 170 bp α-satellite monomers. The red dotted line indicates the monomer size identified by TRASH. The sequence analysed was chromosome 2 92,251,709-94,752,697 bp of CHM13, which contains an α-satellite HOR array. **C.** As for B, but using the ‘--N.max.div’ flag set to 5, which correctly identified the α-satellite monomer length of 170 bp.


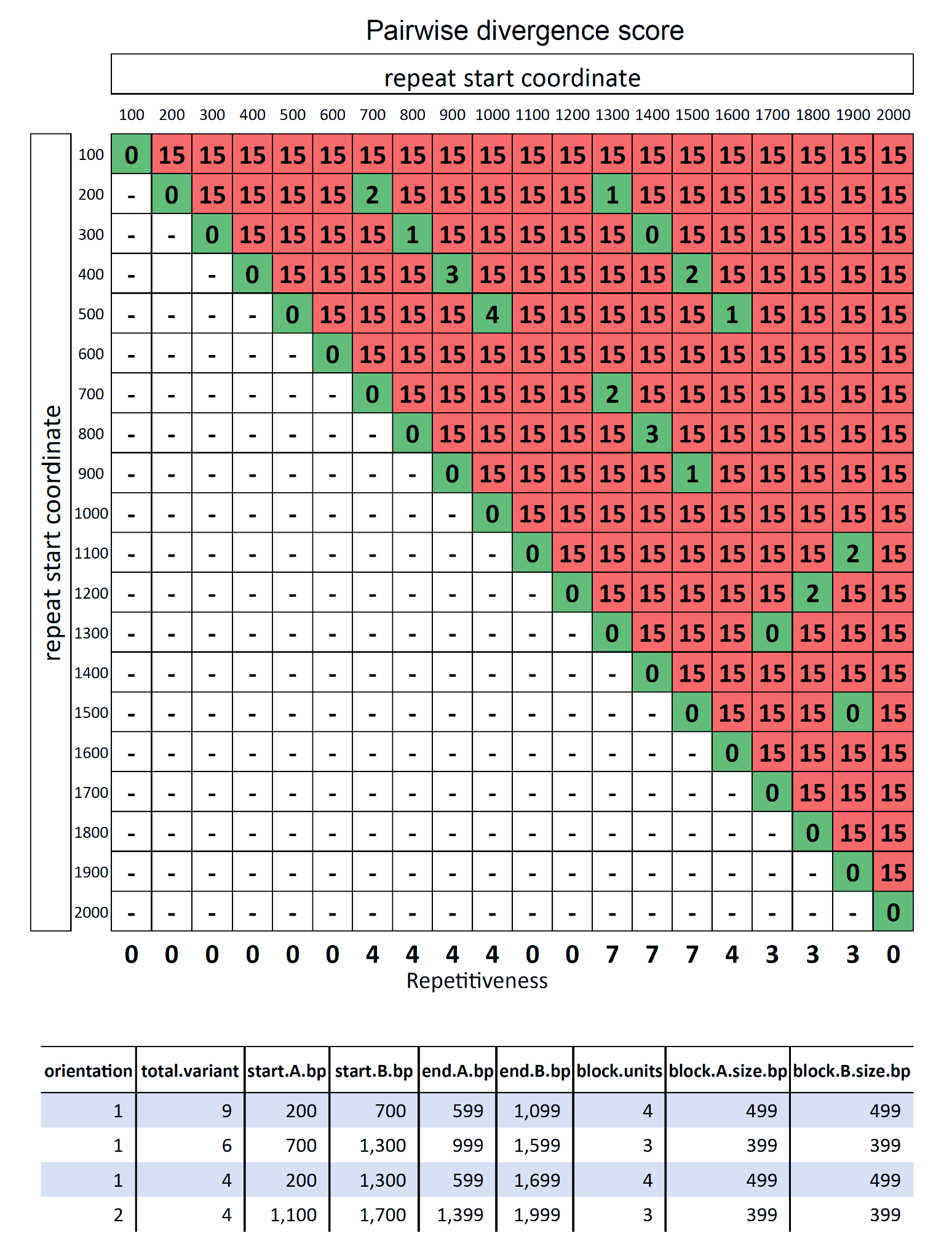


**Supplementary Figure 2. Identification of higher order tandem repeats.** A theoretical region of 20 monomer repeats is shown, with each monomer being 100 bp in length. Pairwise divergence scores between each monomer are shown by the numerical values. When a divergence threshold of 5 is used, green shading is used to show monomer pairs that comprise a higher order repeat (HOR), while red fall above the threshold. Immediately below this matrix, repetitiveness scores for each repeat are shown, which is a sum of all HOR lengths (in monomers) that a repeat is a part of. The table beneath the figure shows a summary of TRASH annotation of the identified HORs. In the first column, ‘1’ represents ‘head-to-tail’ orientation and ‘2’ represents ‘head-to-head’ orientation. ‘Total.variant’ shows the sum of all pairwise divergence scores of the repeat pairs forming the HORs.

**
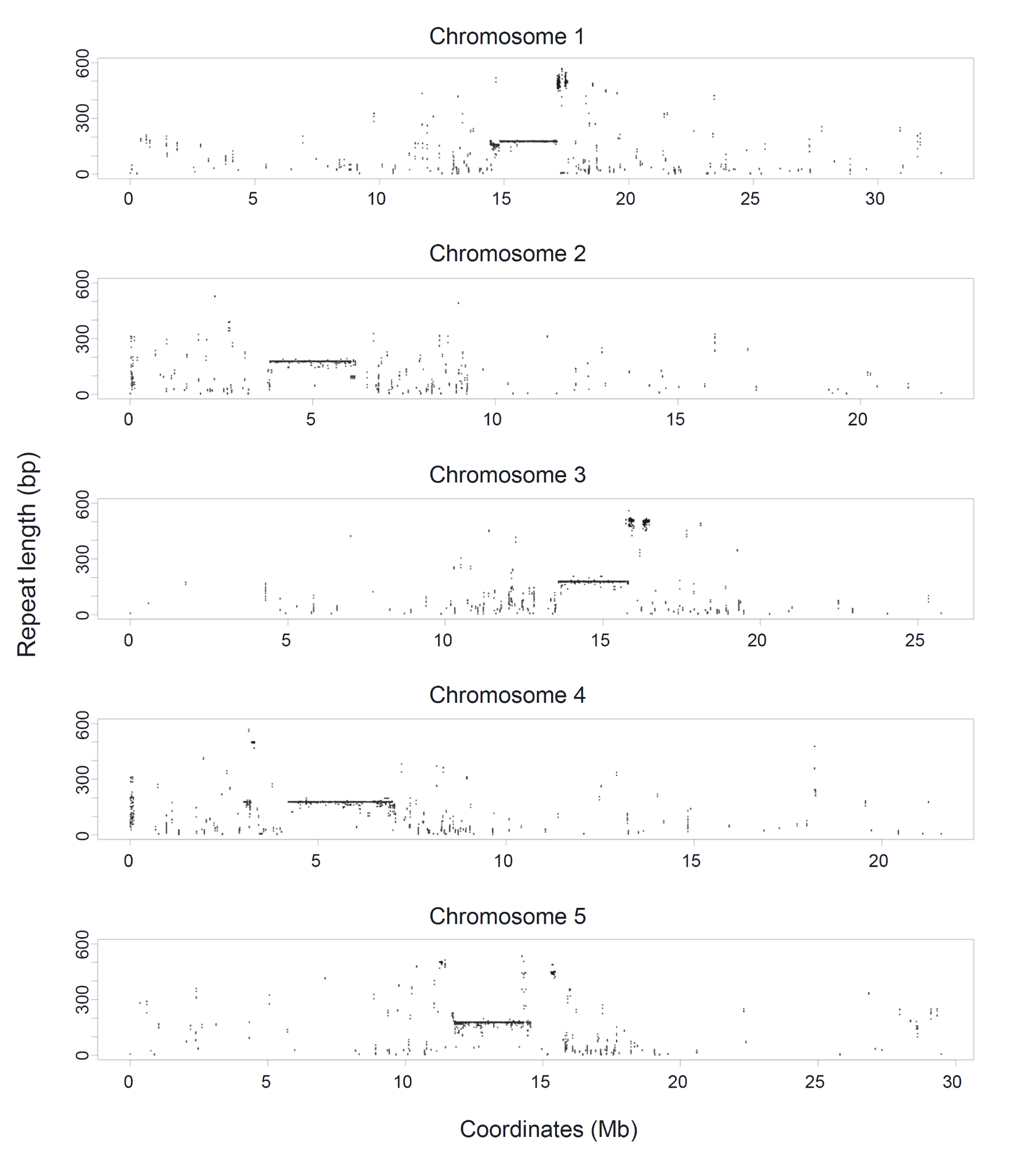
**

**Supplementary Figure 3. Plots of tandem repeat sizes identified in *A. thaliana* Col-CEN assembly using TRASH.** Plots of tandem repeat sizes (bp) are shown along each Col-CEN chromosome. Regions dominated by 178 bp repeats correspond to the centromere locations.

**
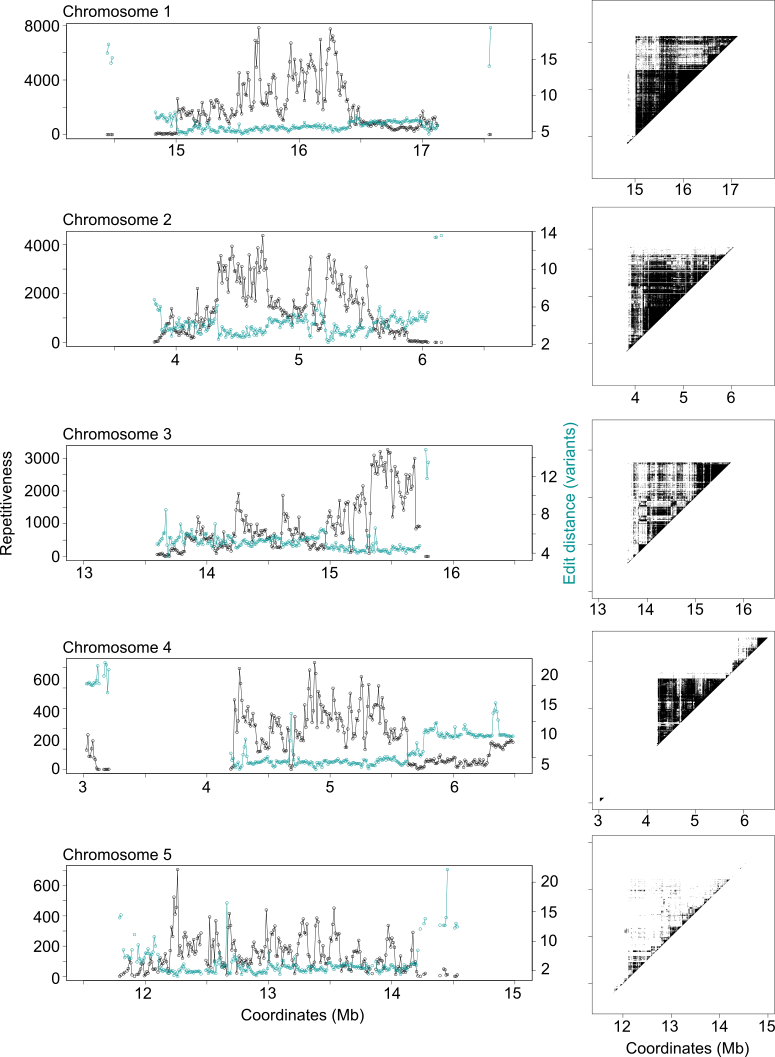
**

**Supplementary Figure 4. Plots of *CEN178* higher order repeats, repetitiveness and edit distance for all chromosomes of the Col-CEN assembly.** For all chromosomes of the *A. thaliana* Col-CEN genome assembly, a dot plot is shown to the right for start positions of blocks of *CEN178* higher order repeats identified by TRASH in the centromere regions. In each case, a subsection of the chromosome is analysed, which contains the centromere repeat arrays. To the left of each dotplot, a moving average plot of *CEN178* HOR repetitiveness (black) and edit distance (blue) averaged over 10 kb adjacent windows are shown for the same regions.


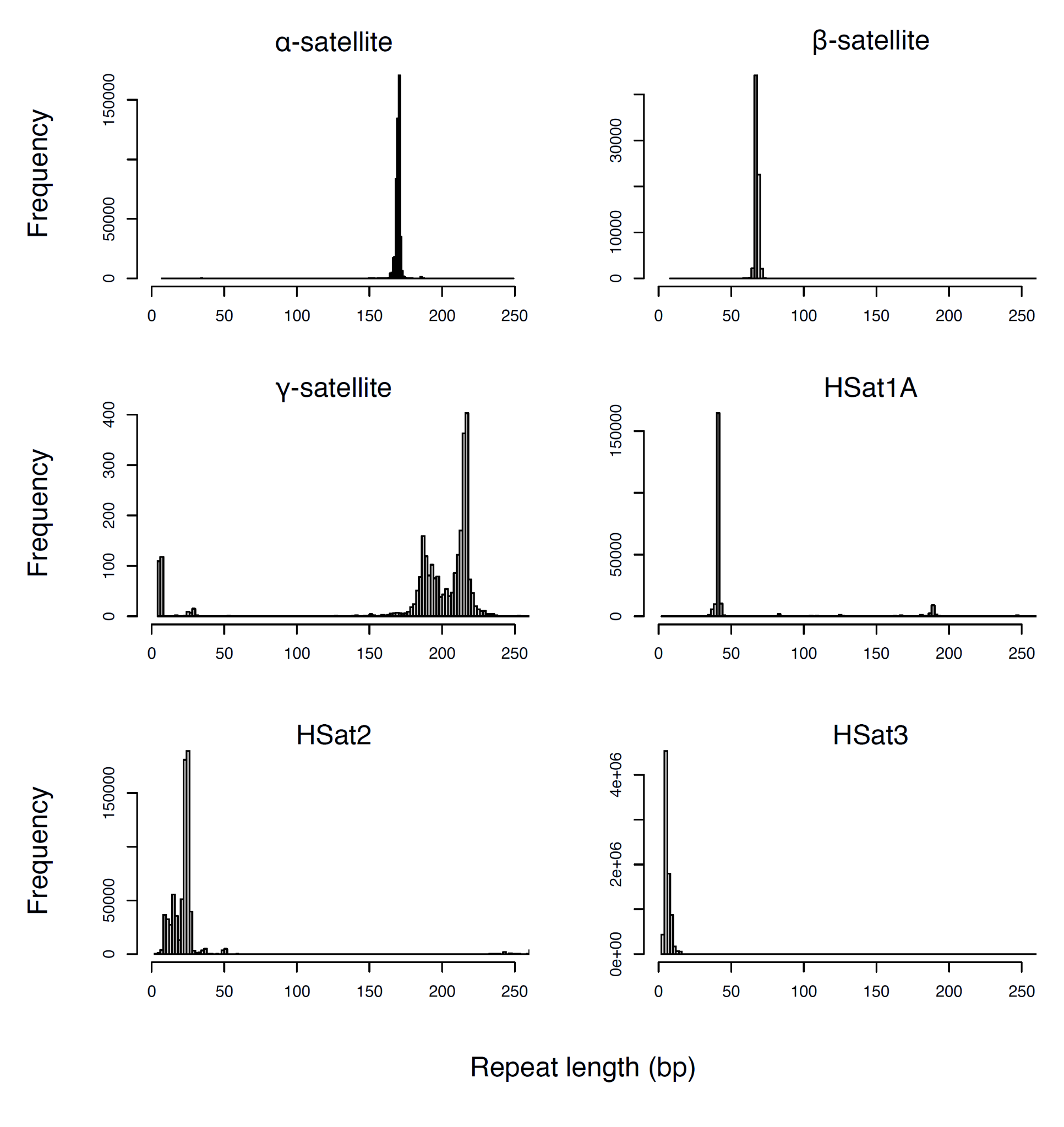


**Supplementary Figure 5. Histograms of human repeat lengths per repeat family.** Human repeats identified by TRASH in *de novo* mode were compared to regions occupied by α-, β- and γ-satellite families, and HSat1A, HSat2 and HSat3, according to Altemose et al [(Altemose *et al.*, 2022)](https://paperpile.com/c/bRhm66/TFc0). Histograms of the lengths of repeats identified by TRASH that overlapped with each family are plotted.

**Supplementary Tables:**

| Sequence | Repeat family name | Length (bp) |
| --- | --- | --- |
| AGTATAAGAACTTAAACCGCAACCGATCTTA  AAAGCCTAAGTAGTGTTTCCTTGTTAGAAGA  CACAAAGCCAAAGACTCATATGGACTTTGGC  TACACCATGAAAGCTTTGAGAAGCAAGAAGA  AGGTTGGTTAGTGTTTTGGAGTCGAATATGA  CTTGATGTCATGTGTATGATTG | *CEN178* | 178 |
| TTGGGAGAAAATGGGTATAAGTGTTGTCTAA  ACACTCCTAATCCATCTCTAACTCTTATAATT  AGTCAAATGCATTGGATTGTGACACATTTTG  ACCATAGAAACACTAACAAAGCTATTTACTG  CTTCTAAGCAATTTTTTGTTGGTTTTAGCCT  CTT | *CEN159* | 159 |
| TCGGAGGGCTGTCTTTGGGCTTTCCGAAAA  GGTATCACATGCCAAGTTTGGCCTCACGGT  CTAAAAGTTATGGAGTCATAAAGTTTTAACC  AAAAAAAAAAAGGTTAAACATAAAAGAGGGA  TGCAACACGAGGACTTCCCGGGAGGTCACC  CATCCTAGTACTACTCTCGCCCAAGCACGC  TTGACTGCGGAGTTCTGATGGGATCCGGT  GCATTAGTGCTGGTATGATCGCATCCGTT  AGTATATGCAATGCAATCGTATATATTCTT  TTTTGAAGACTTGATGAACCATTCGCCGT  GGGTCCCACCCGCTATGTAGGGATACCC  CATCTAGTCTTAACGAGCTTTGATGCATG  AAAAAATTCGAAAACAATGCTTGAACAAG  TAATTTTGGGTCCGTAATATAGCCCAAAT  CACGAAAATGCCCGAAAAAGTACTTAAAG  GTCAAAATTTGGGGTCGACAAAAAGTCAA  TGGAAAAGTTCCATTGTCCTGCTTCTTTCG | *5S* | 502 |

**Supplementary Table 1. Sequence templates used for analysis of the *Arabidopsis thaliana* Col-CEN assembly by TRASH.**

**Supplementary Table 2. Example ‘repeats’ output generated by TRASH on the Col-CEN assembly.** TRASH was run with the HOR module activated and using the *CEN178* family as a template. Due to the large size of the file, the complete table is available for download at: [www.github.com/vlothec/TRASH](http://www.github.com/vlothec/TRASH). Supplementary Table 2 shows the first 20 rows of this table as an example.

**Supplementary Table 3. Example ‘HOR’ output generated by TRASH analysis of the Col-CEN assembly chromosome 2.** The table shows the first 20 rows of TRASH *CEN178* higher order repeat (HORs) annotation of Col-CEN chromosome 2. Output from the remaining table and chromosomes are available at [www.github.com/vlothec/TRASH](http://www.github.com/vlothec/TRASH).

| **Repeat class** | **TRASH (bp)** | **Altemose et al 2022 (bp)** | **% overlap** |
| --- | --- | --- | --- |
| α-Sat | 82,434,207 | 85,669,576 | 96.2 |
| β-Sat | 4,865,095 | 8,609,737 | 56.5 |
| γ-Sat | 502,002 | 650,590 | 77.2 |
| HSat1A | 12,926,921 | 13,390,882 | 96.5 |
| HSat2 | 26,077,409 | 28,705,695 | 90.8 |
| HSat3 | 50,869,161 | 69,334,314 | 73.4 |

**Supplementary Table 4. Overlap of TRASH and published tandem repeat annotation of the human CHM13 genome.** The table compares the total number of tandem repeat base pairs identified by TRASH representing α-, β- and γ- satellite, HSat1A, HSat2 and HSat3 repeats. In addition, the number of bases identified in these repeat classes by Altemose et al is shown and the % overlap with the TRASH annotation [(Altemose *et al.*, 2022)](https://paperpile.com/c/bRhm66/TFc0).

| **Chr** | **α-satellite regions (bp) Altemose 2022** | **TRASH repeats over α-satellite regions (bp)** | **Overlap**  **(%)** | **Identified α-satellite HORs** | **Active HOR Altemose**  **2022** | **TRASH most frequent HOR** |
| --- | --- | --- | --- | --- | --- | --- |
| 1 | 5,202,810 | 5,170,024 | 99.4% | 4,794,345 | 6 | 12 |
| 2 | 2,474,131 | 2,439,058 | 98.6% | 1,514,552 | 4 | 4 |
| 3 | 2,693,578 | 2,609,559 | 96.9% | 125,281 | 17 | 17 |
| 4 | 3,817,023 | 3,795,147 | 99.4% | 753,272 | 19 | 19 |
| 5 | 4,478,762 | 4,243,488 | 94.7% | 1,697,060 | 6 | 4 |
| 6 | 3,389,822 | 3,303,043 | 97.4% | 221,772 | 18 | 18 |
| 7 | 4,754,566 | 4,588,618 | 96.5% | 872,871 | 6 | 6 |
| 8 | 2,778,467 | 2,675,366 | 96.3% | 741,450 | 11 | 7 |
| 9 | 2,985,422 | 2,955,409 | 99.0% | 1,098,615 | 7 | 15 |
| 10 | 2,992,847 | 2,835,550 | 94.7% | 845,141 | 8 | 8 |
| 11 | 4,771,819 | 4,438,988 | 93.0% | 2,597,256 | 5 | 5 |
| 12 | 3,542,720 | 3,386,303 | 95.6% | 1,301,376 | 8 | 8 |
| 13 | 2,698,363 | 2,519,946 | 93.4% | 462,295 | 11 | 7 |
| 14 | 3,997,932 | 3,784,819 | 94.7% | 442,267 | 8 | 8 |
| 15 | 2,796,968 | 2,565,829 | 91.7% | 172,819 | 11 | 20 |
| 16 | 2,670,707 | 2,485,232 | 93.1% | 325,926 | 10 | 10 |
| 17 | 4,354,645 | 4,303,447 | 98.8% | 495,926 | 16 | 16 |
| 18 | 5,582,607 | 5,566,518 | 99.7% | 3,148,028 | 12 | 12 |
| 19 | 5,853,944 | 5,659,182 | 96.7% | 10,918,372 | 6 | 2 |
| 20 | 4,236,992 | 4,092,417 | 96.6% | 225,089 | 16 | 16 |
| 21 | 1,087,204 | 906,302 | 83.4% | 4,201 | 11 | 11 |
| 22 | 4,299,078 | 4,071,080 | 94.7% | 693,291 | 8 | 8 |
| X | 3,719,949 | 3,611,927 | 97.1% | 333,398 | 12 | 12 |
| Y | 489,220 | 426,955 | 87.3% | 410 | 34 | 34 |
|  | | | | | | |

**Supplementary Table 5. α-satellite higher order repeats identified in the human CHM13 genome by TRASH.** The number of base pairs of α-satellites identified in each chromosome of the human CHM13 genome by TRASH and total sizes of α-satellite regions reported in Altemose et al [(Altemose *et al.*, 2022)](https://paperpile.com/c/bRhm66/TFc0), and their % overlap are listed. In addition, the number of α-satellite HORs identified in each centromere array are listed. TRASH was run in *de novo* mode, except for the centromere regions of chromosomes 2, 9, 15 and 21, which were run using the ‘--N.max.div 5’ setting to ensure that α-satellite monomers were identified. The final two columns show the α-satellite ‘Active HOR’ period in monomers identified by Altemose et al [(Altemose *et al.*, 2022)](https://paperpile.com/c/bRhm66/TFc0), and the most common HOR period identified by TRASH. Chromosomes where the TRASH and Altemose HOR periods are matched are highlighted in green.
